# The effect of paternal psoriasis on neonatal outcomes: a nationwide population-based study

**DOI:** 10.3389/fimmu.2023.1172274

**Published:** 2023-04-17

**Authors:** Yu-Huei Huang, Meng-Jiun Chiou, Shun-Fa Yang, Chang-Fu Kuo

**Affiliations:** ^1^ Institute of Medicine, Chung Shan Medical University, Taichung, Taiwan; ^2^ Department of Dermatology, Chang Gung Memorial Hospital, Taoyuan, Taiwan; ^3^ School of Medicine, College of Medicine, Chang Gung University, Taoyuan, Taiwan; ^4^ Division of Rheumatology, Allergy and Immunology, Chang Gung Memorial Hospital, Taoyuan, Taiwan; ^5^ Department of Medical Research, Chung Shan Medical University Hospital, Taichung, Taiwan

**Keywords:** epidemiology, neonatal outcome, pregnancy, psoriasis, risk

## Abstract

**Background:**

Psoriasis is a chronic autoimmune disease involving both environmental and genetic risk factors. Maternal psoriasis often results in poor pregnancies that influence both mothers and newborns. However, the influence of paternal psoriasis on the newborn remains unknown. The aim of this study was to investigate whether paternal psoriasis is associated with increased risk of adverse neonatal outcomes, within a nationwide population-based data setting.

**Methods:**

Singleton pregnancies were identified in the Taiwan National Health Insurance database and National Birth Registry between 2004-2011 and classified into four study groups according to whether mothers and spouses had psoriasis (paternal(−)/maternal(−), paternal(+)/maternal(−), paternal(−)/maternal(+), and paternal(+)/maternal(+)). Data were analyzed retrospectively. Adjusted odds ratios (aOR) or hazard ratios (aHR) were calculated to evaluate the risk of neonatal outcomes between groups.

**Results:**

A total of 1,498,892 singleton pregnancies were recruited. Newborns of fathers with psoriasis but not of mothers with psoriasis were associated with an aHR (95% CI) of 3.69 (1.65–8.26) for psoriasis, 1.13 (1.06–1.21) for atopic dermatitis and 1.05 (1.01–1.10) for allergic rhinitis. Newborns of mothers with psoriasis but not of fathers with psoriasis were associated with an aOR (95% CI) of 1.26 (1.12-1.43) for low birth weight (<2500 g) and 1.64 (1.10–2.43) for low Apgar scores, and an aHR of 5.70 (2.71–11.99) for psoriasis.

**Conclusion:**

Newborns of fathers with psoriasis are associated with significantly higher risk of developing atopic dermatitis, allergic rhinitis and psoriasis. Caution is advised for adverse neonatal outcomes when either or both parents have psoriasis.

## 1 Introduction

Psoriasis is a chronic, T cell-mediated autoinflammatory dermatosis that usually relapses and requires long-term treatment (1–3). It involves both genetic and environmental factors and affects about 2% of the general population (4, 5). Depending on the age of onset, psoriasis has been subclassified into two distinct types: (i) early onset psoriasis, which has onset before the age of 40 years and displays a strong family history (inherited type), comprises about 70% of all psoriasis; and (ii) late onset psoriasis, which has onset at or after age 40 years and is rarely familial (non-inherited type), is more sporadic (6). Maternal immunity is altered during pregnancy, which may increase susceptibility to autoimmune diseases or infections (7). Psoriasis involves aberrant immune responses (1–3), and is associated with a high risk of adverse events in pregnant women with psoriasis (8). Along with several other autoimmune diseases, including inflammatory bowel disease and rheumatoid arthritis, psoriasis has been reported to increase the risk of poor pregnancies (9–11). Reports have also suggested that psoriasis in women was highly associated with the components of metabolic syndrome, including hypertension, abdominal obesity, type 2 diabetes, dyslipidemia and nonalcoholic fatty liver disease (12), which may further deteriorate the clinical status of pregnant women with psoriasis and lead to poor delivery. Previous studies have indicated that maternal psoriasis may increase the risk of adverse maternal and neonatal outcomes (4, 13). Because genetic factors play a strong role in the occurrence of psoriasis, both paternal and maternal psoriasis are likely to contribute to adverse neonatal outcomes.

Positive associations between maternal psoriasis and poor pregnancies have been well demonstrated. Previously we conducted a nationwide population-based study evaluating 2,350,339 singleton pregnancies to investigate the effects of maternal psoriasis on pregnancy and fetal outcomes (13). We found that maternal psoriasis significantly increased the risk of several adverse events including pre-eclampsia, gestational diabetes and hypertension, antepartum hemorrhage, atony during delivery, severe post-partum hemorrhage, cesarean section, stillbirth, fetal prematurity (<37 weeks), low birthweight (<2500 g) of the infant, small gestational age, and low Apgar score. Nevertheless, despite the well-investigated adverse effects of maternal psoriasis on pregnancy and the newborns, studies regarding the neonatal outcomes of paternal psoriasis are very limited. In particular, whether paternal psoriasis has similar effects as maternal psoriasis on infant outcomes remains elusive.

Previous studies have indicated that the inherited type psoriasis displays an autosomal recessive pattern (14, 15). Furthermore, linkage analysis and large population-based studies have suggested parental origin of susceptibility of psoriasis in offspring (5, 16, 17). A cohort study recruiting 103 families with 395 individuals (301 affected, 94 unaffected) (5) found evidence of more profound paternal impact on the occurrence of psoriasis in offspring as more probands had an affected father than an affected mother. Moreover, the authors of that study suggested genetic anticipation because significant intergenerational reduction in age at onset was observed when the psoriasis was inherited from the father. Nevertheless, more comprehensive studies are warranted to investigate associations between psoriasis in both parents and offspring outcomes. The goal of the present study was therefore to determine whether parental psoriasis in both parents or either of the parents alone is associated with increased risk of poor neonatal outcomes, in a nationwide population-based data set.

## 2 Materials and methods

### 2.1 Data sources

This observational cohort study used data from the Taiwan National Health Insurance (NHI) database linked to the National Birth Registry. The NHI database is currently the largest and most accurate population-based nationwide database, and contains the health-related data of 99% of the Taiwan population; it includes comprehensive records of maternity care as well as antenatal examinations (18). All information in the NHI database can be linked to other databases and can be extracted upon request. Researchers were blinded to the personally identifiable information of the subjects. Confidentiality assurances were addressed by complying with the data regulations of the NHI. The National Birth Registry contains information on live births and stillbirths (>20 weeks or 500 g in weight) that has been validated for epidemiological research (19). This also applies to the databases containing demographic data of the pregnant women and their spouses, the pregnancy conditions, and the newborn information and conditions (sex, birth order, gestational age, birthweight, neonatal abnormalities, and Apgar scores).

### 2.2 Ethical considerations

The study protocol was approved by the Institutional Review Board (IRB) of Chang Gung Memorial Hospital (IRB No: 201601155B0). Informed consent of patients was waived because of the retrospective nature of this study and anonymity of data.

### 2.3 Study population

Women with singleton births in Taiwan between 2004 and 2011 whose data were recorded within the NHI database and the National Birth Registry were identified. Among the initially recruited pregnancies, those with stillbirths, absence of neonate’s ID, or mothers aged < 15 years old or > 44 years old, were excluded. The remaining pregnancies were further divided into four study groups based on whether mothers or fathers had psoriasis, as follows: normal (both parents did not have psoriasis, paternal [−]/maternal [−]); fathers had psoriasis while mothers did not have psoriasis (paternal [+]/maternal [−]); fathers did not have psoriasis while mothers had psoriasis (paternal [−]/maternal [+]); both parents had psoriasis (paternal [+]/maternal [+]). Clinical determination of psoriasis was according to the International Classification of Disease, ninth revision (ICD-9-CM), clinical modification code 696.1. This study only recruited adult patients who had undergone at least two consensus diagnoses of psoriasis within one year by qualified dermatologists or rheumatologists.

### 2.4 Outcomes

Pregnancy and neonatal outcomes were identified from the National Birth Registry. The maximum observational term for newborns was 12 years, till December 2015. Adverse neonatal outcomes included infant death, low birth weight (<2500 g), preterm (< 37 weeks), low Apgar scores (at 5 min < 7), atopic dermatitis (ICD-9-CM: 691.88), allergic rhinitis (ICD-9-CM: 477.0, 477.1, 477.2, 477.8, 477.9), asthma (ICD-9-CM: 493.xx), rheumatoid arthritis (ICD-9-CM: 714.0), autism (ICD-9-CM: 299.0), psoriasis (ICD-9-CM: 696.1), systemic lupus erythematosus (ICD-9-CM: 710.0), and cleft palate (ICD-9-CM: 749.xx).

### 2.5 Covariates

Maternal and paternal covariates included age groups (<20, 20–29, 30–39, ≥ 40 years) and Charlson comorbidity index (CCI) values. Additional maternal covariates included smoking and alcohol consumption, as well as maternal country of origin. Comorbidity identified for CCI included myocardial infarction, congestive heart failure, peripheral vascular disease, cerebrovascular disease, dementia, chronic pulmonary disease, rheumatologic disease, ulcer disease, mild liver diseases, diabetes with or without chronic complications, hemiplegia or paraplegia, renal disease, any malignancy (including leukemia and lymphoma), moderate or severe liver disease, metastatic solid tumor, and HIV infection. Neonate covariates included sex, birthweight, and gestational age. The validated versions of ICD-9 codes were used, as previously described by Deyo et al. (20).

### 2.6 Statistical analysis

The subjects were divided into four study groups based on paternal and/or maternal psoriasis as described above. The generalized estimating equation (GEE) model was used to estimate odds ratios (ORs) and corresponding 95% confidence intervals (CI) for each outcome between the four study groups. Correlation analysis was based on an autoregressive model. Odds ratios (ORs) and hazard ratios (HRs) were adjusted for confounders, including maternal and paternal age, sex of neonates, maternal and paternal CCI, and maternal nationality. A two-tailed test with 5% level of significance was used for all statistical hypotheses. All statistical analysis was performed using SAS version 9.3 statistics software (SAS Institute, Cary, NC, USA).

## 3 Results

### 3.1 Demographic characteristics

**Figure 1:**
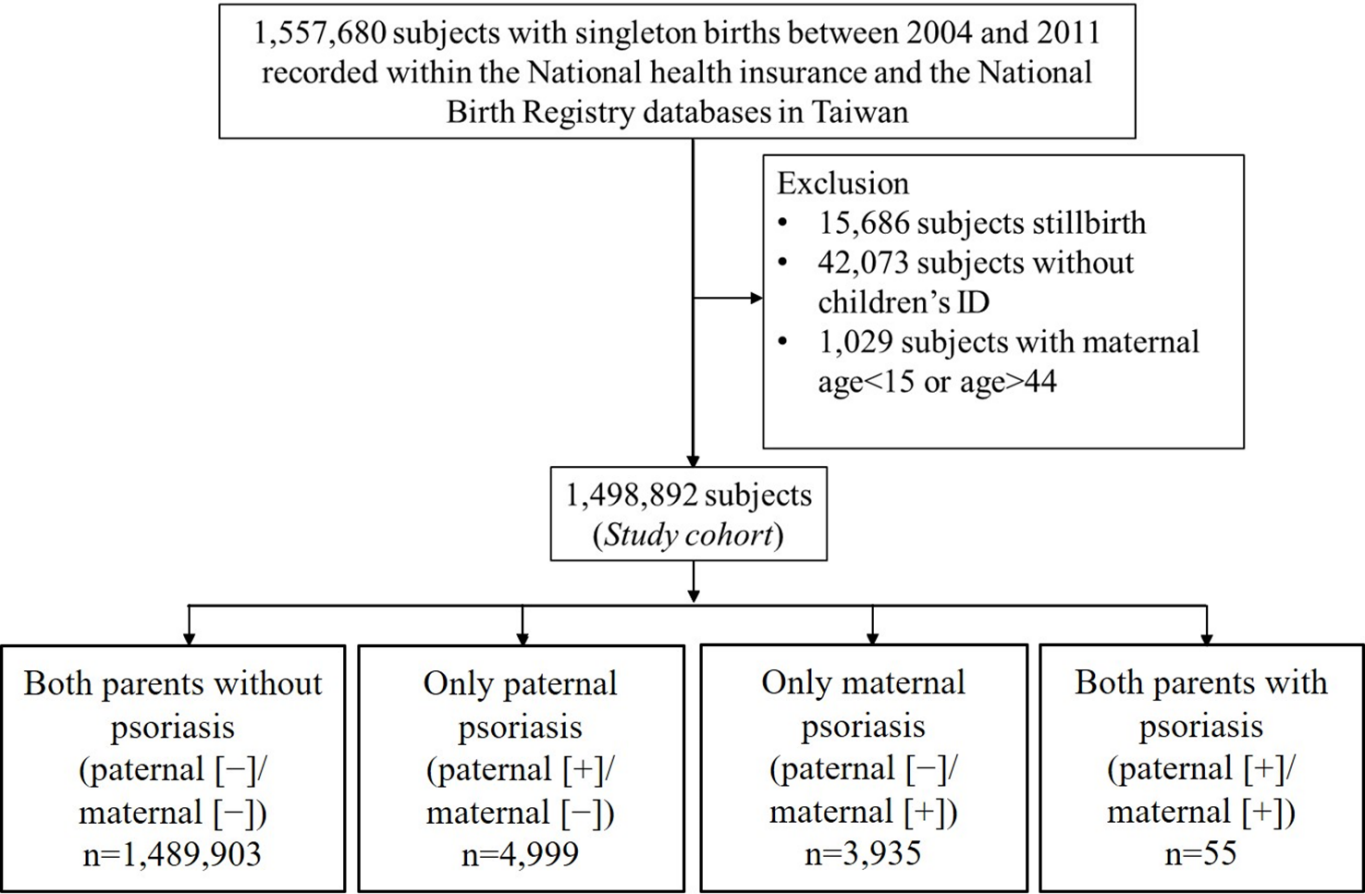
Flowchart of the population-based retrospective cohort study.

Initially, 1,557,680 women who had singleton births in Taiwan between 2004 and 2011 were identified in the NHI database and the National Birth Registry. The flow chart of the data retrieval process is illustrated in **Figure 1**. After excluding those who had stillbirths (n=15,686), absence of children’s ID (n=42,073), or who were aged < 15 years or > 44 years (n=1,029), data of 1,498,892 delivery records were finally retrieved and analyzed retrospectively. According to maternal and paternal psoriasis, subjects were divided into 4 groups as follows: paternal (−)/maternal (−) (n=1,489,903); paternal (+)/maternal (−) (n=4,999); paternal (−)/maternal (+) (n=3,935); paternal (+)/maternal (+) (n=55). Comparison of baseline demographic and clinical characteristics between the four groups is shown in **Table 1**.

**Table 1:** Baseline demographic and clinical characteristics of pregnancies between parents with or without psoriasis.

Characteristic	paternal [−]/maternal [−] (n = 1,489,903)	paternal [+]/maternal [−] (n=4,999)	paternal [−]/maternal [+] (n=3,935)	paternal [+]/maternal [+] (n=55)	P value
**Maternal age, mean (SD), y**	29.59 (4.80)	30.55 (4.52)	30.56 (4.30)	31.71 (3.76)	<0.001*
<20	33,084 (2.22)	51 (1.02)	37 (0.94)	0 (0.00)	
20–29	752,768 (50.52)	2,173 (43.47)	1,681 (42.72)	19 (34.55)	
30–39	680,186 (45.65)	2,664 (53.29)	2,166 (55.04)	36 (65.45)	
≧40	23,865 (1.60)	111 (2.22)	51 (1.30)	0 (0.00)	
**Paternal age, mean (SD), y**	33.04 (5.48)	34.18 (5.60)	33.12 (5.01)	35.14 (4.77)	<0.001*
<20	4,728 (0.32)	2 (0.04)	5 (0.13)	0 (0.00)	
20–29	408,469 (27.42)	1,050 (21.00)	990 (25.16)	9 (16.36)	
30–39	862,059 (57.86)	3,280 (65.61)	2,441 (62.03)	37 (67.27)	
≧40	142,462 (9.56)	667 (13.34)	330 (8.39)	9 (16.36)	
**Male infant, n (%)**	778,586 (52.26)	2,616 (52.33)	2,033 (51.66)	29 (52.73)	0.904
**Smoking in pregnancy (mother), n (%)**	1,405 (0.09)	2 (0.04)	1 (0.03)	0 (0.00)	0.494
**Drinking in pregnancy (mother), n (%)**	260 (0.02)	0 (0.00)	2 (0.05)	0 (0.00)	0.794
**Foreign nationals (mother), n (%)**	151,876 (10.19)	569 (11.38)	46 (1.17)	1 (1.82)	<0.001*
Maternal comorbidity					
Charlson comorbidity index, mean (SD)	0.10 (0.39)	0.12 (0.42)	0.17 (0.50)	0.15 (0.40)	<0.001*
Myocardial infarct, n (%)	47 (0.00)	0 (0.00)	0 (0.00)	0 (0.00)	0.963
Congestive heart failure, n (%)	939 (0.06)	5 (0.10)	8 (0.20)	0 (0.00)	0.004*
Peripheral vascular disease, n (%)	1,242 (0.08)	2 (0.04)	10 (0.25)	0 (0.00)	0.001*
Cerebrovascular disease	2,325 (0.16)	4 (0.08)	9 (0.23)	0 (0.00)	0.351
Dementia, n (%)	209 (0.01)	0 (0.00)	0 (0.00)	0 (0.00)	0.738
Chronic pulmonary disease, n (%)	34,310 (2.30)	122 (2.44)	126 (3.20)	1 (1.82)	0.002*
Rheumatologic disease, n (%)	10,949 (0.73)	38 (0.76)	93 (2.36)	1 (1.82)	<0.001*
Ulcer disease, n (%)	65,095 (4.37)	272 (5.44)	207 (5.26)	1 (1.82)	<0.001*
Mild liver disease, n (%)	4,232 (0.28)	20 (0.40)	21 (0.53)	0 (0.00)	0.011*
Diabetes without chronic complication, n (%)	7,550 (0.51)	29 (0.58)	48 (1.22)	0 (0.00)	<0.001*
Diabetes with chronic complications, n (%)	1,525 (0.10)	6 (0.12)	11 (0.28)	0 (0.00)	0.007*
Hemiplegia or paraplegia, n (%)	353 (0.02)	2 (0.04)	1 (0.03)	0 (0.00)	0.902
Renal disease, n (%)	555 (0.04)	3 (0.06)	1 (0.03)	0 (0.00)	0.835
Any malignancy including leukemia and lymphoma, n (%)	5,003 (0.34)	20 (0.40)	28 (0.71)	0 (0.00)	0.001*
Moderate or severe liver disease, n (%)	115 (0.01)	0 (0.00)	0 (0.00)	0 (0.00)	0.875
Metastatic solid tumor, n (%)	324 (0.02)	0 (0.00)	1 (0.03)	0 (0.00)	0.452
HIV infection, n (%)	314 (0.02)	3 (0.06)	0 (0.00)	0 (0.00)	0.219
Paternal comorbidity					
Charlson comorbidity index, mean (SD)	0.13 (0.46)	0.21 (0.62)	0.14 (0.50)	0.20 (0.56)	<0.001*
Myocardial infarct, n (%)	572 (0.04)	4 (0.08)	5 (0.13)	0 (0.00)	0.017*
Congestive heart failure, n (%)	2,302 (0.15)	19 (0.38)	12 (0.30)	0 (0.00)	<0.001*
Peripheral vascular disease, n (%)	2,041 (0.14)	11 (0.22)	8 (0.20)	0 (0.00)	0.281
Cerebrovascular disease, n (%)	4,190 (0.28)	19 (0.38)	10 (0.25)	0 (0.00)	0.574
Dementia, n (%)	318 (0.02)	2 (0.04)	3 (0.08)	0 (0.00)	0.137
Chronic pulmonary disease, n (%)	33,445 (2.24)	177 (3.54)	98 (2.49)	3 (5.45)	<0.001*
Rheumatologic disease, n (%)	4,941 (0.33)	70 (1.40)	10 (0.25)	0 (0.00)	<0.001*
Ulcer disease, n (%)	76,745 (5.15)	338 (6.76)	209 (5.31)	3 (5.45)	<0.001*
Mild liver disease, n (%)	15,534 (1.04)	95 (1.90)	37 (0.94)	1 (1.82)	<0.001*
Diabetes without chronic complication, n (%)	13,796 (0.93)	86 (1.72)	38 (0.97)	1 (1.82)	<0.001*
Diabetes with chronic complications, n (%)	3,958 (0.27)	17 (0.34)	11 (0.28)	0 (0.00)	0.750
Hemiplegia or paraplegia, n (%)	683 (0.05)	2 (0.04)	1 (0.03)	0 (0.00)	0.994
Renal disease, n (%)	1,643 (0.11)	7 (0.14)	7 (0.18)	0 (0.00)	0.556
Any malignancy, including leukemia and lymphoma, n (%)	5,290 (0.36)	35 (0.70)	12 (0.30)	1 (1.82)	<0.001*
Moderate or severe liver disease, n (%)	482 (0.03)	2 (0.04)	4 (0.10)	0 (0.00)	0.117
Metastatic solid tumor, n (%)	355 (0.02)	3 (0.06)	2 (0.05)	0 (0.00)	0.433
HIV infection, n (%)	108 (0.01)	0 (0.00)	1 (0.03)	0 (0.00)	0.542

HIV, Human Immunodeficiency Virus; SD, standard deviation. *p<0.05.

The mean ages of the mothers at the time of delivery and the fathers were significantly different between the four study groups (both P<0.0001). Maternal comorbidities with significant differences between the groups included congestive heart failure (P=0.0041), peripheral vascular disease (P=0.001), chronic pulmonary disease (P=0.0019), rheumatologic disease (P<0.0001), gastric ulcer disease (P<0.0001), mild liver disease (P=0.0112), diabetes without chronic complications (P<0.0001), diabetes with chronic complications (P=0.0068), and malignancies (P=0.0006). Paternal comorbidities with significant between-group differences included myocardial infarction (P=0.017) and malignancies (P=0.0001). Both maternal and paternal CCI values were significantly different between the four study groups.

### 3.2 Parents psoriasis and fetal-neonatal outcomes

**Table 2** illustrates the adjusted ORs (aORs) of adverse neonatal outcomes, including stillbirth, low birth weight (< 2500 g), preterm (< 37 weeks) and Apgar scores at 5 min of < 7 min. Neonates in the group of paternal (−)/maternal (+) had generally poorer outcomes. GEE analysis revealed that the ORs of low birth weight were 1.26 (95% CI 1.12–1.43) times higher and the ORs of low Apgar score were 1.64 (95% CI 1.10–2.43) times higher in the group of paternal (−)/maternal (+). No significantly increased odds were observed for stillbirth, low birth weight, preterm and low Apgar scores in paternal (+)/maternal (−) and paternal (+)/maternal (+) groups.

**Table 2:** Comparison of fetal outcomes between pregnancies associated with maternal and paternal psoriasis or not.

	n (incidence rate, per 1000 person-years)				Crude Odds Ratio (95% CI)				Adjusted Odds Ratio^a^ (95% CI)			
Variables	paternal [−]/maternal [−] (n = 1,489,903)	paternal [+]/maternal [−] (n=4,999)	paternal [−]/maternal [+] (n=3,935)	paternal [+]/maternal [+] (n=55)	paternal [−]/maternal [−] (n=1,489,903)	paternal [+]/maternal [−] (n=4,999)	paternal [−]/maternal [+] (n=3,935)	paternal [+]/maternal [+] (n=55)	paternal [−]/maternal [−] (n=1,489,903)	paternal [+]/maternal [−] (n=4,999)	paternal [−]/maternal [+] (n=3,935)	paternal [+]/maternal [+] (n=55)
Infant death	5,890 (3.95)	14 (2.80)	11 (0.28)	1 (1.82)	Reference	0.71 (0.40-1.24)	0.70 (0.39-1.27)	4.79 (0.66-34.70)	Reference	0.77 (0.44-1.34)	0.72 (0.40-1.31)	5.29 (0.75-37.5)
Low birth weight (<2500 g)	86,737 (58.22)	260 (52.01)	292 (7.42)	4 (7.27)	Reference	0.90 (0.79-1.02)	1.30 (1.15-1.47)*	1.20 (0.36-3.98)	Reference	0.94 (0.83-1.07)	1.26 (1.12-1.43)*	1.23 (0.37-4.08)
Preterm (<37 week)	105,374 (70.73)	333 (66.61)	301 (7.65)	3 (5.45)	Reference	0.94 (0.84-1.05)	1.09 (0.96-1.23)	0.83 (0.26-2.62)	Reference	0.95 (0.85-1.07)	1.05 (0.93-1.19)	0.82 (0.26-2.59)
APGAR SCORE at 5 min <7	6,131 (4.12)	19 (3.80)	26 (0.66)	2 (3.64)	Reference	0.93 (0.59-1.45)	1.66 (1.12-2.47)*	4.54 (0.63-32.90)	Reference	0.99 (0.63-1.56)	1.64 (1.10-2.43)*	4.71 (0.65-34.0)

a Adjusted0 for maternal age, paternal age, infant sex, maternal Charlson comorbidity index, paternal Charlson comorbidity index, maternal nationality.

*p<0.05.

95%CI: 95%Confidence interval; APGAR, Appearance, Pulse, Grimace, Activity, and Respiration.

### 3.3 Parents’ psoriasis and long-term offspring outcomes

Complications in the neonates are illustrated in **Table 3**. Most complications were not significantly associated with parents’ psoriasis except that neonates in the paternal (+)/maternal (−) group were significantly associated with atopic dermatitis (adjusted HR [aHR] [95% CI] of 1.13 [1.06–1.21]), and with allergic rhinitis (aHR [95% CI] of 1.05 [1.01–1.10]). Psoriasis in neonates was significantly associated with paternal (+)/maternal (−) and paternal (−)/maternal (+) (aHR [95% CI] of 3.69 [1.65–8.26] and 5.70 [2.71–11.99]), respectively. None of the investigated complications were significantly associated with the paternal (+)/maternal (+) group.

**Table 3:** Comparison of long-term fetal outcomes between pregnancies associated with maternal and paternal psoriasis or not.

	n (incidence rate, per 1000 person-years)				Crude Hazard Ratio (95% CI)				Adjusted Hazard Ratio^a^ (95% CI)			
Variables	paternal [−]/maternal [−] (n = 1,489,903)	paternal [+]/maternal [−] (n=4,999)	paternal [−]/maternal [+] (n=3,935)	paternal [+]/maternal [+] (n=55)	paternal [−]/maternal [−] (n=1,489,903)	paternal [+]/maternal [−] (n=4,999)	paternal [−]/maternal [+] (n=3,935)	paternal [+]/maternal [+] (n=55)	paternal [−]/maternal [−] (n=1,489,903)	paternal [+]/maternal [−] (n=4,999)	paternal [−]/maternal [+] (n=3,935)	paternal [+]/maternal [+] (n=55)
Atopic dermatitis	272,744 (26.41)	1,036 (32.72)	789 (31.66)	13 (38.88)	Reference	1.16 (1.09-1.24)*	1.12 (1.04-1.20)*	1.36 (0.76-2.44)	Reference	1.13 (1.06-1.21)*	1.07 (0.99-1.15)	1.29 (0.72-2.31)
Allergic rhinitis	695,220 (82.73)	2,395 (92.00)	1,859 (90.77)	23 (75.21)	Reference	1.08 (1.04-1.13)*	1.06 (1.01-1.11)*	0.9 (0.58-1.41)	Reference	1.05 (1.01-1.10)*	1.02 (0.97-1.07)	0.87 (0.55-1.36)
Asthma	458,486 (46.91)	1,541 (49.99)	1,211 (50.38)	11 (30.69)	Reference	1.03 (0.97-1.09)	1.03 (0.97-1.09)	0.64 (0.32-1.3)	Reference	1.01 (0.96-1.07)	1.00 (0.94-1.07)	0.62 (0.31-1.27)
Rheumatoid arthritis	179 (0.01)	0 (0.00)	0 (0.00)	0 (0.00)	Reference	NA	NA	NA	Reference	NA	NA	NA
Autism	10,955 (0.91)	34 (0.90)	25 (0.85)	1 (2.49)	Reference	0.96 (0.69-1.35)	0.9 (0.61-1.34)	2.68 (0.4-18.04)	Reference	0.92 (0.66-1.29)	0.87 (0.59-1.29)	2.54 (0.38-6.96)
Psoriasis	534 (0.04)	6 (0.16)	7 (0.24)	0 (0.00)	Reference	3.74 (1.67-8.37)*	5.68 (2.7-11.93)*	NA	Reference	3.69 (1.65-8.26)*	5.70 (2.71-11.99)*	NA
Systemic lupus erythematosus	287 (0.02)	1 (0.03)	2 (0.07)	0 (0.00)	Reference	2.32 (0.58-9.34)	1.52 (0.21-10.78)	NA	Reference	2.25 (0.56-9.05)	1.39 (0.20-9.86)	NA
Cleft palate	2,695 (0.22)	9 (0.24)	10 (0.34)	0 (0.00)	Reference	1.01 (0.52-1.94)	1.42 (0.76-2.65)	NA	Reference	1.03 (0.53-1.98)	1.42 (0.76-2.65)	NA

a Adjusted for maternal age, paternal age, infant sex, maternal Charlson comorbidity index, paternal Charlson comorbidity index, maternal nationality.

*p<0.05.

95%CI: 95%Confidence interval; NA, not applicable.

## 4 Discussion

The present study was the first to elucidate the clinical effects of paternal psoriasis on neonatal outcomes within a nationwide, population-based database. Results of the present study revealed that paternal psoriasis significantly increased the risks of neonatal psoriasis, atopic dermatitis and allergic rhinitis compared with those in normal parents, while no significant differences were found in stillbirth, low birth weight, preterm birth, and low Apgar scores. Maternal psoriasis was also observed to significantly increase the risks of low birth weight, low Apgar scores and newborn psoriasis, which agreed with our previous findings and other observations (13, 21, 22). However, results for both parents with psoriasis were not significantly associated with adverse neonatal outcomes. Results also suggest that paternal psoriasis may play a role in poorer long-term neonatal outcomes (atopic dermatitis, allergic rhinitis, psoriasis).

Maternal psoriasis is well established as often leading to poorer pregnancies (4, 13, 21–23). Previously, in a nationwide population-based study in Taiwan between 2001 and 2012, we revealed that maternal psoriasis was strongly associated with several adverse pregnancy events, including pre-eclampsia, gestational diabetes, gestational hypertension, antepartum hemorrhage, atony, severe post-partum hemorrhage, and Cesarean delivery; as well as adverse neonatal outcomes, including stillbirth (with both known and unexplained reasons), prematurity, low birth weight and low Apgar scores (13). Similarly, the present study showed that neonatal low birth weight and low Apgar scores were significantly associated with maternal psoriasis but not in paternal psoriasis. Psoriasis is a cutaneous inflammatory disease with dysregulated T-cell response and pro-inflammatory cytokine release (24). These cytokines have also been found to be increased in maternal serum or in the cord blood and may have harmful effects on the placenta, thus leading to preterm birth and SGA neonates (25). Notably, the present study expanded these findings and further examined the associations between either parent or both parents’ psoriasis with other adverse neonatal outcomes, including atopic dermatitis, allergic rhinitis, asthma, rheumatoid arthritis, autism, systemic lupus erythematosus and cleft palate. Among these, we found that both the paternal (+)/maternal (−) and paternal (−)/maternal (+) groups of parents were significantly associated with increased risk of neonatal psoriasis, and that the paternal (+)/maternal (+) group was not significantly associated with most of these adverse neonatal outcomes. This implies that, although maternal psoriasis may result in some adverse pregnancy events, certain long-term adverse outcomes may not occur in the neonates.

Psoriasis and atopic dermatitis have been viewed as distinct diseases. The first onset of atopic dermatitis usually occurs in infancy or early childhood, while psoriasis occurs sporadically in young children but has its peak onset in late adolescent or early adulthood (26). Psoriasis is mainly driven by activation of Th17 T-cells and the associated secretion of IL-17. Atopic dermatitis, in contrast, is believed to be mediated by Th2 and Th22, and the associated secretion of IL-4/IL-13 and IL-22, respectively (26). Despite such distinction between the two diseases, however, studies have indicated that some specific subtypes of atopic dermatitis, including the Asian-origin and pediatric atopic dermatitis, are highly associated with activation of Th17 and increased secretion of IL-17 and thus have some overlap with the histopathology of psoriasis (27). Atopic dermatitis, allergic rhinitis and asthma are among the most common childhood atopic diseases and share similar underlying aberrantly activated immune mediators (28–31). Individual genetic susceptibility may also play a role in these outcomes (28). On the other hand, epidemiological studies have shown that psoriasis is also associated with allergic rhinitis (30) and asthma (32), although the underlying molecular mechanisms remain unclear. Nevertheless, evidence has implied that shared immune mediators may be involved. Allergic rhinitis and asthma were once thought to be exclusively mediated by Th2. However, just like atopic dermatitis, these are also heterogeneous disorders with multiple immunological phenotypes, involving blending of Th17 activation, the main mediator of psoriasis (30). Interestingly, the present study showed that only the paternal (+)/maternal (−) group of patients of psoriasis were significantly associated with atopic dermatitis and allergic rhinitis in the offspring. Based on these findings, we hypothesized that paternal psoriasis imposes more profound effects on these associated atopic diseases than maternal psoriasis. Currently, studies regarding associations between paternal psoriasis and atopic dermatitis and allergic rhinitis are limited, and our results have shed light on the possibility of such associations. Nevertheless, a more comprehensive, large population-based genetic link analysis is required to further elucidate this hypothesis.

The group of paternal (+)/maternal (+) of patients had a 5.29-fold higher risk for stillbirth, a 1.23-fold higher risk for low birth weight, a 4.71-fold higher risk for low Apgar score, a 1.29-fold higher risk for atopic dermatitis and a 2.54-fold higher risk for autism, but no significant differences were found between these associations. Notably, no psoriasis occurred in the offspring of this group of patients. However, the lack of statistical significance for these adverse neonatal outcomes and zero events for neonatal psoriasis may be due to the small sample size of this group. In addition, we cannot rule out the possibility that some patients in this group had relatively mild psoriasis, therefore it was not significantly associated with adverse outcomes of the newborns. According to Yang et al., severe maternal psoriasis is associated with higher risk of low birth weight as compared with mild maternal psoriasis [4], consequently, rare or no symptoms may occur in the offspring when psoriasis is mild in the parents. However, we cannot exclude the possibility that complications may occur later in life, and studies with longer follow-up are required to investigate this issue.

It has been reported that maternal psoriasis and low maternal socioeconomic position may increase the rate of offspring psoriasis (33). Further, the present study demonstrated that maternal psoriasis is an independent risk factor of offspring psoriasis. Additional evidence indicates that genetic factors affect psoriasis susceptibility (34). Twin studies can distinguish genetic factors from shared environmental and unique environmental factors. The classic twin design assumes that monozygotic (MZ) twins share not only all their genes but also their upbringing and early environment, while dizygotic (DZ) twins share on average half of their segregating genes in addition to their upbringing and early environment. Previous twin studies have shown a higher concordance rate of psoriasis in MZ than in DZ twins (34), which supports a genetic effect on psoriasis.

Although psoriasis can be a hereditary disease and has an estimated heritability of 66% (35), the parent-of-origin effect in transmission remains questionable. In a cohort study that included 640 patients, the authors indicated that a sex-specific epigenetic inheritance pattern for psoriasis was not observed. Moreover, no significant differences were noted in the clinical profiles of the disease between patients grouped by transmission pattern of psoriasis (36). However, in another large cohort study that recruited 849 patients (16), a greater chance of an increase in psoriasis severity was noted when psoriasis was transmitted by an affected father compared to an affected mother, and a significantly larger proportion of the probands were reported to have an affected father, suggesting a paternal transmission bias for psoriasis. In addition, further evidence has been provided through linkage analysis studies that have identified genetic loci possibly involved in paternal transmission of psoriasis (5, 17). In contrast, the present nationwide, population-based clinical data study revealed that paternal psoriasis was significantly associated with increased risks of offspring psoriasis and atopic diseases, which may further support the concept of paternal transmission of psoriasis. Nevertheless, our findings cannot determine how profoundly the father and mother influence psoriasis in the offspring, because both paternal and maternal psoriasis are significantly associated with psoriasis in the newborn.

The present study has several limitations. First, despite comprehensive validation of the records in the NHI database and National Birth Registry, the possibility of misclassification of psoriasis could not be excluded. Second, the definition of the population is based on the eligibility of the databases, which did not include data on pregnancies with less than 21 gestational weeks or an abortus whose weight was less than 500 g. Third, the number of neonates in the paternal (+)/maternal (+) group was few which led to difficulty in interpreting their effect on the offspring outcomes. Lastly, data about the fathers’ environmental factors and clinical severity of psoriasis, such as air pollutant, psoriasis with or without psoriatic arthritis, was not available in the NHI database, therefore we were unable to assess the associations of disease severity with the risk of adverse neonatal outcomes.

## 5 Conclusions

The results found that most of offspring were not significantly associated with parental psoriasis, except for atopic dermatitis and allergic rhinitis in the paternal (+)/maternal (-) group, and psoriasis in neonates with parental psoriasis. These findings suggest that parental psoriasis may increase the risk of long-term offspring outcome, and further research is needed to understand the underlying mechanisms and potential interventions to reduce this risk.

## Data availability statement

The original contributions presented in the study are included in the article/supplementary material. Further inquiries can be directed to the corresponding author.

## Ethics statement

The studies involving human participants were reviewed and approved by Chang Gung Memorial Hospital. The ethics committee waived the requirement of written informed consent for participation.

## Author contributions

Y-HH, S-FY and C-FK had made substantial contributions to the conception and study design. Y-HH and C-FK collected the data. M-JC analyzed the data. Y-HH prepared the manuscript, Y-HH, M-JC, S-FY and C-FK critical revision of the manuscript. Y-HH Funding acquisition. All authors contributed to the article and approved the submitted version.

## References

[1] Rendon A Schäkel K. Psoriasis pathogenesis and treatment. Int J Mol Sci (2019) 20(6):1475. doi: 10.3390/ijms20061475 PMC6471628 30909615

[2] Boehncke WH Schön MP. Psoriasis. Lancet (2015) 386(9997):983–94. doi: 10.1016/S0140-6736(14)61909-7 26025581

[3] Liang Y Sarkar MK Tsoi LC Gudjonsson JE. Psoriasis: a mixed autoimmune and autoinflammatory disease. Curr Opin Immunol (2017) 49:1–8. doi: 10.1016/j.coi.2017.07.007 28738209 PMC5705427

[4] Yang YW Chen CS Chen YH Lin HC. Psoriasis and pregnancy outcomes: a nationwide population-based study. J Am Acad Dermatol (2011) 64(1):71–7. doi: 10.1016/j.jaad.2010.02.005 20970879

[5] Burden AD Javed S Bailey M Hodgins M Connor M Tillman D. Genetics of psoriasis: paternal inheritance and a locus on chromosome 6p. J Invest Dermatol (1998) 110(6):958–60. doi: 10.1046/j.1523-1747.1998.00213.x 9620305

[6] Queiro R Tejon P Alonso S Coto P. Age at disease onset: a key factor for understanding psoriatic disease. Rheumatol (Oxford) (2014) 53(7):1178–85. doi: 10.1093/rheumatology/ket363 24273020

[7] Jamieson DJ Theiler RN Rasmussen SA. Emerging infections and pregnancy. Emerging Infect diseases (2006) 12(11):1638–43. doi: 10.3201/eid1211.060152 PMC3372330 17283611

[8] Bobotsis R Gulliver WP Monaghan K Lynde C Fleming P. Psoriasis and adverse pregnancy outcomes: a systematic review of observational studies. Br J Dermatol (2016) 175(3):464–72. doi: 10.1111/bjd.14547 26991866

[9] Reed SD Vollan TA Svec MA. Pregnancy outcomes in women with rheumatoid arthritis in Washington state. Maternal Child Health J (2006) 10(4):361–6. doi: 10.1007/s10995-006-0073-3 16649008

[10] Dominitz JA Young JC Boyko EJ. Outcomes of infants born to mothers with inflammatory bowel disease: a population-based cohort study. Am J gastroenterol (2002) 97(3):641–8. doi: 10.1111/j.1572-0241.2002.05543.x 11926208

[11] Mahadevan U Sandborn WJ Li DK Hakimian S Kane S Corley DA. Pregnancy outcomes in women with inflammatory bowel disease: a large community-based study from northern California. Gastroenterology (2007) 133(4):1106–12. doi: 10.1053/j.gastro.2007.07.019 17764676

[12] Gisondi P Fostini AC Fossà I Girolomoni G Targher G. Psoriasis and the metabolic syndrome. Clinics Dermatol (2018) 36(1):21–8. doi: 10.1016/j.clindermatol.2017.09.005 29241748

[13] Huang YH Yee Ng C Chiou MJ Kuo CF. Fetal-neonatal and maternal outcomes in women with psoriasis vulgaris: a nationwide population-based registry linkage study in Taiwan. J Dermatol (2020) 48(2):184–9. doi: 10.1111/1346-8138.15658 33205465

[14] Swanbeck G Inerot A Martinsson T Wahlström J. A population genetic study of psoriasis. Br J Dermatol (1994) 131(1):32–9. doi: 10.1111/j.1365-2133.1994.tb08454.x 8043420

[15] Swanbeck G Inerot A Martinsson T Enerbäck C Enlund F Samuelsson L. Genetic counselling in psoriasis: empirical data on psoriasis among first-degree relatives of 3095 psoriatic probands. Br J Dermatol (1997) 137(6):939–42. doi: 10.1046/j.1365-2133.1997.19892070.x 9470911

[16] Pollock RA Thavaneswaran A Pellett F Chandran V Petronis A Rahman P. Further evidence supporting a parent-of-Origin effect in psoriatic disease. Arthritis Care Res (2015) 67(11):1586–90. doi: 10.1002/acr.22625 26017758

[17] Karason A Gudjonsson JE Upmanyu R Antonsdottir AA Hauksson VB Runasdottir EH. A susceptibility gene for psoriatic arthritis maps to chromosome 16q: evidence for imprinting. Am J Hum Genet (2003) 72(1):125–31. doi: 10.1086/345646 PMC378616 12474146

[18] NHI. Universal health coverage in Taiwan: national health insurance administration national health insurance administration: national health insurance administration(2020). Available at: https://www.nhi.gov.tw/english/Content_List.aspx?n=8FC0974BBFEFA56D&topn=ED4A30E51A609E49.

[19] Lin CM Lee PC Teng SW Lu TH Mao IF Li CY. Validation of the Taiwan birth registry using obstetric records. J Formosan Med Assoc (2004) 103(4):297–301. 15175826

[20] Deyo RA Cherkin DC Ciol MA. Adapting a clinical comorbidity index for use with ICD-9-CM administrative databases. J Clin Epidemiol (1992) 45(6):613–9. doi: 10.1016/0895-4356(92)90133-8 1607900

[21] Lima XT Janakiraman V Hughes MD Kimball AB. The impact of psoriasis on pregnancy outcomes. J Invest Dermatol (2012) 132(1):85–91. doi: 10.1038/jid.2011.271 21918537

[22] Cohen-Barak E Nachum Z Rozenman D Ziv M. Pregnancy outcomes in women with moderate-to-severe psoriasis. J Eur Acad Dermatol Venereology JEADV (2011) 25(9):1041–7. doi: 10.1111/j.1468-3083.2010.03917.x 21108670

[23] Ben-David G Sheiner E Hallak M Levy A. Pregnancy outcome in women with psoriasis. J Reprod Med (2008) 53(3):183–7. 18441722

[24] Bonifati C Carducci M Cordiali Fei P Trento E Sacerdoti G Fazio M. Correlated increases of tumour necrosis factor-alpha, interleukin-6 and granulocyte monocyte-colony stimulating factor levels in suction blister fluids and sera of psoriatic patients–relationships with disease severity. Clin Exp Dermatol (1994) 19(5):383–7. doi: 10.1111/j.1365-2230.1994.tb02687.x 7955493

[25] Sorokin Y Romero R Mele L Wapner RJ Iams JD Dudley DJ. Maternal serum interleukin-6, c-reactive protein, and matrix metalloproteinase-9 concentrations as risk factors for preterm birth <32 weeks and adverse neonatal outcomes. Am J Perinatol (2010) 27(8):631–40. doi: 10.1055/s-0030-1249366 PMC2976602 20195952

[26] Guttman-Yassky E Krueger JG. Atopic dermatitis and psoriasis: two different immune diseases or one spectrum? Curr Opin Immunol (2017) 48:68–73. doi: 10.1016/j.coi.2017.08.008 28869867

[27] Brunner PM Guttman-Yassky E Leung DY. The immunology of atopic dermatitis and its reversibility with broad-spectrum and targeted therapies. J Allergy Clin Immunol (2017) 139(4S):S65–76. doi: 10.1016/j.jaci.2017.01.011 PMC5405702 28390479

[28] Moreno MA. Atopic diseases in children. JAMA Pediatrics (2016) 170(1):96–. doi: 10.1001/jamapediatrics.2015.3886 26747075

[29] Lee E Hong SJ. Phenotypes of allergic diseases in children and their application in clinical situations. Korean J Pediatrics (2019) 62(9):325–33. doi: 10.3345/kjp.2018.07395 PMC6753312 31096745

[30] Galili E Barzilai A Twig G Caspi T Daniely D Shreberk-Hassidim R. Allergic rhinitis and asthma among adolescents with psoriasis: a population-based cross-sectional study. Acta Derm Venereol (2020) 100(10):adv00133. doi: 10.2340/00015555-3485 32314795 PMC9137373

[31] Guttman-Yassky E Krueger JG Lebwohl MG. Systemic immune mechanisms in atopic dermatitis and psoriasis with implications for treatment. Exp Dermatol (2018) 27(4):409–17. doi: 10.1111/exd.13336 28266782

[32] Fang HY Liao WC Lin CL Chen CH Kao CH. Association between psoriasis and asthma: a population-based retrospective cohort analysis. Br J Dermatol (2015) 172(4):1066–71. doi: 10.1111/bjd.13518 25385450

[33] Groot J Nybo Andersen AM Adam A Tind Nielsen TE Blegvad C Skov L. Associations between maternal socioeconomic position and psoriasis: a cohort study among the offspring of the Danish national birth cohort. Br J Dermatol (2019) 180(2):321–8. doi: 10.1111/bjd.17091 30117154

[34] Lonnberg AS Skov L Skytthe A Kyvik KO Pedersen OB Thomsen SF. Heritability of psoriasis in a large twin sample. Br J Dermatol (2013) 169(2):412–6. doi: 10.1111/bjd.12375 23574549

[35] Grjibovski AM Olsen AO Magnus P Harris JR. Psoriasis in Norwegian twins: contribution of genetic and environmental effects. J Eur Acad Dermatol Venereology JEADV (2007) 21(10):1337–43. doi: 10.1111/j.1468-3083.2007.02268.x 17958839

[36] Di Lernia V Ficarelli E Lallas A Ricci C. Familial aggregation of moderate to severe plaque psoriasis. Clin Exp Dermatol (2014) 39(7):801–5. doi: 10.1111/ced.12401 25156221

